# Kelvin–Voigt Parameters Reconstruction of Cervical Tissue-Mimicking Phantoms Using Torsional Wave Elastography

**DOI:** 10.3390/s19153281

**Published:** 2019-07-25

**Authors:** Antonio Callejas, Antonio Gomez, Inas H. Faris, Juan Melchor, Guillermo Rus

**Affiliations:** 1 Department of Structural Mechanics, University of Granada, 18071 Granada, Spain; 2 Instituto de Investigación Biosanitaria, ibs.GRANADA, 18012 Granada, Spain; 3 Department of Mechanical Engineering, University College London, London WC1E 6BT, UK; 4 Excellence Research Unit, “Modelling Nature” (MNat), University of Granada, 18071 Granada, Spain

**Keywords:** Torsional Wave Elastography, Shear Wave Elastography, tissue-mimicking phantom, viscoelasticity, Kelvin-Voigt model, cervical biomechanics

## Abstract

The reconstruction of viscous properties of soft tissues, and more specifically, of cervical tissue is a challenging problem. In this paper, a new method is proposed to reconstruct the viscoelastic parameters of cervical tissue-mimicking phantoms by a Torsional Wave Elastography (TWE) technique. The reconstruction method, based on a Probabilistic Inverse Problem (PIP) approach, is presented and experimentally validated against Shear Wave Elastography (SWE). The anatomy of the cervical tissue has been mimicked by means of a two-layer gelatine phantom that simulates the epithelial and connective layers. Five ad hoc oil-in-gelatine phantoms were fabricated at different proportion to test the new reconstruction technique. The PIP approach was used for reconstructing the Kelvin-Voigt (KV) viscoelastic parameters by comparing the measurements obtained from the TWE technique with the synthetic signals from a Finite Difference Time Domain (FDTD) KV wave propagation model. Additionally, SWE tests were realized in order to characterize the viscoelastic properties of each batch of gelatine. Finally, validation was carried out by comparing the KV parameters inferred from the PIP with those reconstructed from the shear wave dispersion curve obtained from the SWE measurements. In order to test the degree of agreement between both techniques, a Student’s T-test and a Pearson’s correlation study were performed. The results indicate that the proposed method is able to reconstruct the KV viscoelastic properties of the cervical tissue, for both the epithelial and connective layers, as well as the thickness of the first layer with acceptable accuracy.

## 1. Introduction

The cervix is a muscular part of the female reproductive system located at the lower end of the uterus. The cervix plays a crucial role during the gestation, acting as a gatekeeper, supporting the fetus inside the uterus and keeping it safe from external hazard [1]. From the moment of conception biological transformations in the cervix happen until the childbirth. These transformations, triggered by mechanical and chemical processes, have an impact in the physiology of the cervical tissue to the point of altering its mechanical properties [2]. For instance, before and during the delivery, the cervical structure is deformed to allow the descent of the baby into the birth canal [3]. The mechanical transformation during gestation consists in a decrease of stiffness of the cervix [2,4,5]. This mechanical transformation is linked to changes of the structural collagen, which mainly are the reduction in the cross-linking between fibers and the shift of the distinctive arrangement of the fibers, from mostly aligned to curled [6].

The preterm birth is the first cause of infant mortality and morbidity in children under five years of age [7,8,9]. The change in the mechanical properties of the cervical tissue before and during the delivery provides the basis for being used as an indicator of the preterm birth [10], so treatment actions can be taken as anticipation of the preterm birth. One of the most employed techniques is SWE imaging for quantify tissue softness or stiffness in cervical tissue [4,5,11,12,13,14]. Limitations of this technique in the cervical tissue are: first, it is highly attenuating due to its microstructural complexity and second the deposited energy in the medium [15,16]. Alternative dynamic techniques based on torsional vibrations were employed in the characterization of soft tissue viscoelastic properties. Valtorta et al. [17] proposed a new method for measuring the complex shear modulus of soft biological tissues using a torsional resonator. In vitro experiments on bovine and porcine liver demonstrated the feasibility of the technique. Henni et al. [18] presented shear wave induced resonance technique for dynamic ultrasound elastography of confined mechanical inclusions. The method relies on torsional shear waves modeled with the Helmholtz equation in spherical coordinates. This approach was validated with in vivo measurements on a breast fibroadenoma. A more recent study carried out by Yengul et al. [19] analyzed the dispersion in tissue-mimicking gels using a combination of SWE with a torsional vibration rheometry. The precision of the results of torsional vibration rheometry was comparable with SWE. A new promising elastography technique (TWE) was proposed for measuring the viscoelastic properties of cervical tissue [20,21]. The technique employs a transvaginal probe that makes contact with the cervix for transmitting and receiving a torsional wave propagated through tissue. The use of a torsional wave presents the advantage of isolating a pure shear motion, thus, eliminating the generation of spurious compressional waves [20,21].

To the date of this work, the method that TWE uses for reconstructing the viscoelastic properties is based on a time-of-flight (TOF) approach [21]. The TWE probe transmits and receives a torsional wave that not only propagates along the surface of the cervix but also in depth. Therefore, the wave not only interacts with the most superficial layer, the epithelium, but also with the immediately below layer made of connective tissue. The TOF-based method is blind to the fact that the cervix is composed of several layers and that these layers have different mechanical properties. The integrity of the cervical tissue is ensured by the extracellular matrix, where its histological composition provides support of the structural functionality. The basic composition of the matrix is a cross-linked network of collagen fibers enclosed by a ground substance. In the cervix, the collagen is mainly divided into two types I (67%) and III (33%), a proportion that is maintained throughout the entire pregnancy [22,23,24]. Regarding the epithelial layer, no content of collagen fibers was reported in literature [25]. Taking into account that the content and distribution of collagen define the strength of the tissue, a new approach with different layers of the cervix is required. The technique used in this work for the reconstruction of the viscoelastic parameters was based on a numerical model that simulated the propagation of shear waves through the phantom and that did not only take into account the start of the signal but the entire received signal was analyzed.

In this work, a probabilistic approach, based on a new metric of information density and a numerical forward wave propagation model of torsional waves, is proposed for solving the probabilistic inverse problem (PIP) [26]. The final aim is the reconstruction of the viscoelastic properties of the layers that form the cervical tissue structure. Methods based on the use of a forward wave propagation model within an optimization algorithm for reconstructing the viscoelastic properties of soft tissue can be found in the literature, for instance, the deterministic inverse problem approach using a genetic algorithm [27,28] for prostate cancer detection. The reconstruction of the parameters has several limitations if one considers the existence of noise in the measurements performed, heterogeneity in the properties of the sample, and even the fact that the model used to simulate its behavior is an approximation of reality. To solve these limitations, the use of a probabilistic approach based on the theory of Tarantola [29] was employed [26,30].

The wave propagation model requires a constitutive law that defines the stress-strain relationship of the cervical tissue. Although the commercial elastography techniques consider a pure elastic relationship, it is well known that the mechanical response of soft tissue is viscoelastic [28,31,32]. Viscosity has an important role in wave propagation by increasing attenuation and producing frequency dispersion. Furthermore, viscosity is becoming an interesting parameter to study, since it might provide additional characterization of pathologies [33]. According to the results of the work carried out by Callejas et al. [21] the Kelvin-Voigt model was the simplest model that best fit the stress-strain relationship of the ex-vivo human cervical tissue when using the TWE technique. The proposed wave propagation model is numerically developed using a finite difference time domain (FDTD) algorithm. The choice was made from a practical point of view since solving the torsional wave propagation analytically is complex and, after considering that the algorithm offers simplicity, speed and flexibility for implementing a singular constitutive law such as the Kelvin-Voigt (KV) model.

Validation is a crucial step when using numerical models for simulating physical phenomena, such is the propagation of torsional waves in this case. Validation can be achieved by different means, for instance by comparing the observations from experimental work against the computational model. Well-accepted experimental techniques can be used to measure certain features of the physical phenomenon that can be compared with model simulations. The model is validated when a certain level of agreement is reached. For instance, Gómez Fernández [28] used experimental observations from rheometry and high-speed camera in translucent prostate phantoms for validating the numerical wave propagation model. In this case, the numerical simulations using the FDTD wave propagation model were compared against results from experimental tests in multi-layer cervix-like gelatine phantoms using the well-established SWE technique by the Verasonics system (Verasonics Inc., Kirkland, WA, USA).

Therefore, the main aim of this work is to propose a new method for the reconstruction of KV viscoelastic parameters in cervical tissue-mimicking phantoms using the TWE technique. For such a purpose, this paper has been divided into several sections. First, a brief introduction to the core content has been shown. Second, a section of materials and methodology details the methods used in this work. A probabilistic approach was employed to reconstruct the KV viscoelastic parameters by comparing the results obtained from TWE technique with the synthetic signals from the FDTD KV model. Additionally, the characterization of the phantoms was realized by SWE measurements, the gold standard technique in elastography. Third, the results of the comparison between the experimental tests using SWE and the numerical simulations were tested using Student’s T-test and a Pearson’s correlation study. Finally, sections for the discussion of the results and conclusion of the work are shown.

## 2. Materials and Methods

**Figure 1:**
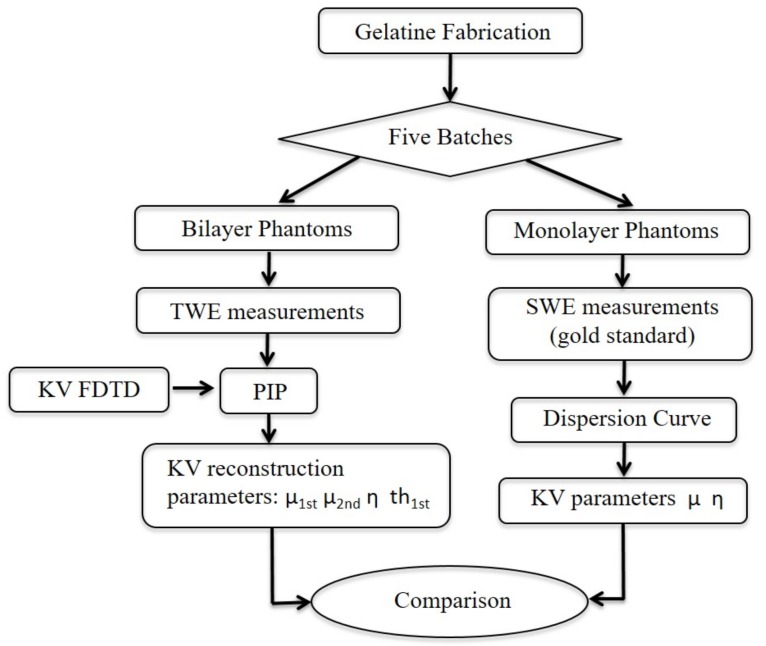
Schematic flowchart used for validating the KV viscoelastic model.

The proposed methodology consists of five steps (see Figure 1): (1) An idealization of the mechanical behaviour of the cervical tissue with five different tissue-mimicking phantoms; (2) A viscoelastic characterization of the phantoms using SWE (single-layer phantoms); (3) The measurement of the phantoms using the TWE technique (bilayer phantoms); (4) A PIP is performed to reconstruct the phantom viscoelastic parameters by comparing the experimental signals using the TWE technique and the synthetic signals from the FDTD KV model; (5) finally, comparison between the parameters obtained with TWE technique and SWE is analyzed.

### 2.1. Tissue-Mimicking Phantom Fabrication

The viscoelastic characterization of the cervical tissue requires the use of a model that simulates the torsional wave propagation. According to the results of the work carried out by Callejas et al. [21], the KV model is the simplest model that best fit the ex-vivo cervical tissue experimental results. The next step after selecting the model is its validation, which consists of measuring tissue-mimicking phantoms with TWE technique and comparing against SWE, a well-known technique by the scientific community.

Fabrication of tissue-mimicking phantoms is straightforward in general and has been described by taking into account characteristics from the recipe by [34]. After studying different ingredients and fabrication methods, the ingredients found in Table 1 were proposed. Potassium sorbate was included to keep the phantom from early decay due to bacterial and fungal activity. The formalin ingredient was used to raise the melting point of the gelatine, stabilizing the response of the phantom properties to room temperature variations and as a cross-linking agent. Finally, sodium dodecyl sulfate (surfactant) was added for a better mixture of oil with water. Phantoms contain different concentrations of gelatine and oil to mimic the elastic and viscous parameters of cervical tissue. In addition, the constituent oil generates scattering for the visualization of the shear wave propagation through the phantom with SWE technique. Three different gelatine percentages were considered (7.5, 10 and 15 %) as well as two percentages of oil (5 and 10 %).

**Figure 2:**
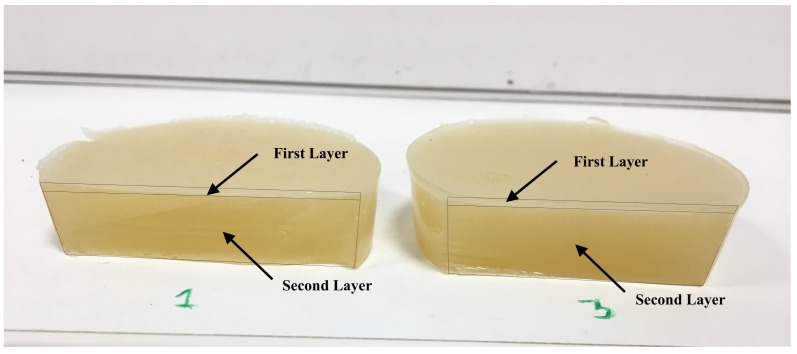
Phantoms number 1 and 3. The phantoms were unmolded and cut to appreciate the two layers of which they are composed.

Taking into account the nature of the cervical tissue, composed of epithelial and connective layers, the phantoms were fabricated considering two layers (see Figure 2). The specific percentages of gelatine, oil and the thickness of the first layer (simulating the epithelial layer of the cervix) have been chosen to simulate the viscoelastic properties of the cervical tissue according to the evidence found in the literature [35,36,37]. For this purpose, five different phantoms have been fabricated. The ingredients that interfere with the parameters of the KV model are those that have been varied (gelatine, oil and layer thickness), the rest were kept constant (formalin, K-sorbate, surfactant and $H_{2}O$). The aim is to vary each of the parameters of each phantom that are reconstructed and verify that the PIP is capable of reconstructing them. Details about the percentages of gelatine and oil, as well as the thickness of the two layers are given in Table 2.

The procedure carried out for the fabrication of the phantoms required for measurements with TWE technique is listed below. The phantoms for the measurements with SWE technique have been manufactured with the same batches for the TWE phantoms but in round molds of larger diameter (7 cm) and greater depth (6 cm) for correct measurement with the above technique. The steps followed to fabricate the phantoms are based on the procedure followed by Dunmire et al. [34].

1. Weigh and prepare each of the components listed in Table 1.
2. Add the K-sorbate to the distilled water and begin mixing for five min.
3. Add the surfactant and keep mixing for another 5 min.
4. Add the oil with the previous solution and mix manually at a rate that minimized the formation of air bubbles and the formation of large clumps.
5. Heat the combined solution at a rate of approximately 1 °C per minute.
6. Gradually add the gelatine powder to the combined solution.
7. Allow 5–10 min to verify that the gelatine is well mixed.
8. Cover and heat the solution to 85 °C at a rate of approximately 1 °C per minute.
9. Hold the solution between 85 °C and 90 °C for 90 min.
10. Cool the mixture from 85 °C to 40 °C at a rate of 1 °C per minute.
11. Add the formalin and mix the solution for 5 min.
12. Pour the mixture into the round molds (5 cm in diameter) until the thickness of the connective layer is reached. A thickness of 15 mm has been considered to avoid interference in the measurements due to reflections with the bottom of the gelatin.
13. Wait until the mixture reaches 37 °C–38 °C.
14. While the first batch is getting cold, repeat steps 1 to 11 with the ingredients needed to fabricate the first layer.
15. When both batches reach the temperature of 37 °C–38 °C, carefully pour the second batch (first layer) on the first batch using a syringe. According to the dimensions of the mold, the volume of gelatine necessary to reach the required thickness is calculated.
16. Leave the phantom to solidify at room temperature for 2 h before being stored in the refrigerator.
17. Remove the phantom from the refrigerator and left at room temperature for 6 h.

### 2.2. SWE Characterization of the Phantoms

Mechanical properties of soft tissue are directly related to the speed of the waves propagating through it [38,39,40,41]. A perturbation in the media is needed in order to capture shear waves. In this work, Acoustic Radiation Force (ARF) is used to excite the tissue producing its deformation; tissue displacements are generated due to this focused ARF and induce shear waves that propagate away from this push.

**Figure 3:**
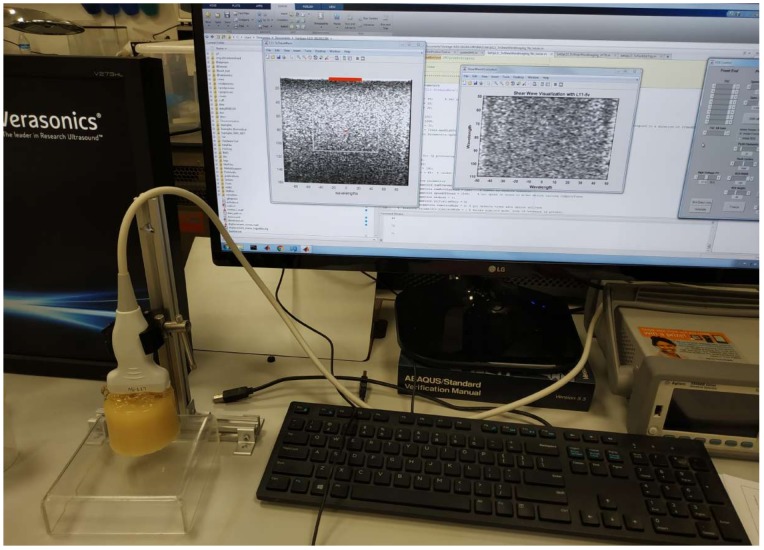
Experimental setup of Verasonics measurements.

A programmable research ultrasound system (Vantage, 128 Verasonics Inc., Kirkland, WA, USA) was configured to provide both the B-mode image and the shear wave motion speed. A linear-array transducer with a center frequency of 7.8 MHz and properties shown in Table 3 was used for imaging (L11−5v, Verasonics Inc.). Each of the five phantoms was imaged three times and shear wave speeds were estimated. All the measurements were performed at laboratory temperature (22 ± 1 °C). Verasonics offers big flexibility in sequence design and permits access to raw data from each element of the array [42]. Verasonics uses the MATLAB programming environment (Release 2018b, MathWorks, Natick, United States), the user needs to write a program script to generate the imaging sequence. Several types of pushing sequences can be used to generate shear waves, in this work Multiple Track Location (MTL) SWE Imaging is used for a single focused push for the center of the frequency of 7.8 MHz. Scanning process of a gelatin phantom with the Verasonics system is shown in Figure 3.

In Table 4, the SWE acquisition parameters used in this paper are shown. Push transmit frequency and track frequency are typically selected to be the center frequency of the transducer. However, in this work, lower track transmit frequency was used to successfully receive the harmonic frequency in the case of harmonic tracking. Furthermore, for push transmit frequency, a lower frequency was also used to minimize underestimation of tracked tissue displacement due to speckle shearing within the track point spread function. In this paper, we used a push frequency that is in the lower −25 dB bandwidth of the transducer to expand the push beam keeping a high transmit efficiency [43,44].

#### 2.2.1. Tissue Motion Estimation

**Figure 4:**
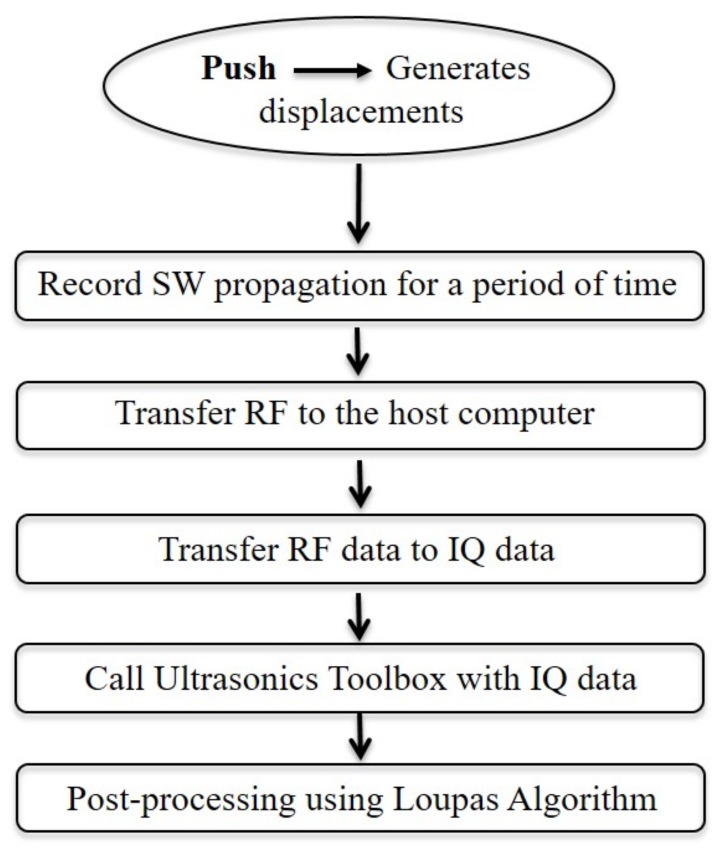
Schematic flowchart used for postprocessing IQ data.

The Loupas algorithm was used to estimate the axial displacements [45]. This is done by postprocessing the In-phase and Quadrature (IQ) data obtained from the shear wave propagation as follows (see Figure 4):

- Generate the push sequence (Create displacements).
- Transmit shear waves according to the push sequence.
- Record shear wave propagation during a period of time.
- Transfer the recorded RF to the host computer, then transform RF data to IQ data.
- Call the Ultrasound Toolbox (USTB) with the IQ data and post-process using Loupas 2D autocorrelator.

Although Verasonics offers postprocessing of IQ data using the Kasai algorithm it is recommended to use Loupas 2D autocorrelator. This is because Kasai’s algorithm uses a constant frequency to compute the phase shift: the center frequency of the transducer. However, the center frequency of RF-echoes decreases along with the axial depth, and Loupas approach corrects the mean RF frequency along with each axial extent and this leads to getting more accurate results. In this paper post-processing of the IQ data measured was done using the USTB [46].

#### 2.2.2. Dispersion Shear Wave Speed Curve

From the speed field resulted from SWE excitation, the dispersion shear wave speed curve was obtained for each batch of single-layer phantom. Using the curve as a reference, the viscoelastic tissue properties can be extracted by fitting the data with a mechanical model of the tissue. For a linear, elastic, isotropic, homogeneous and unbounded material, the shear wave speed ($c_{s}$) is expressed in terms of the shear elasticity μ and density ρ by the relation:

(1) $$c_{s}=\sqrt{\frac{\mu }{\rho }}$$

In contrast, for a viscoelastic material described by the KV model, the shear wave speed is not a constant value, it depends on the angular frequency (ω) [31,32].

(2) $$c_{s}\left(\omega \right)=\sqrt{\frac{2(\mu^{2}+\omega^{2}\eta^{2})}{\rho (\mu +\sqrt{\mu^{2}+\omega^{2}\eta^{2}})}}$$

where η is the KV shear viscosity of the sample. The density of soft tissue is usually assumed to be 1000 $\mathrm{kg}/m^{3}.$

This dispersion, shown in the frequency-dependent phase velocity and shear attenuation is the consequence of the propagation of the shear waves through the tissue [38]. The phase shear wave speed was obtained using the equation:

(3) $$c_{s}\left(\omega \right)=\frac{\omega \Delta r}{\Delta \phi (\omega )}$$

A Fast Fourier Transform (FFT) of the tissue speed field has been used to obtain the phase change Δϕ(r,t) of the wave over the traveled distance Δr at each frequency [47,48]. Finally, the dispersion curve obtained was fitted by Equation (2) to get the shear elasticity (μ) and the shear viscosity (η).

### 2.3. TWE Characterization of the Phantoms

**Figure 5:**
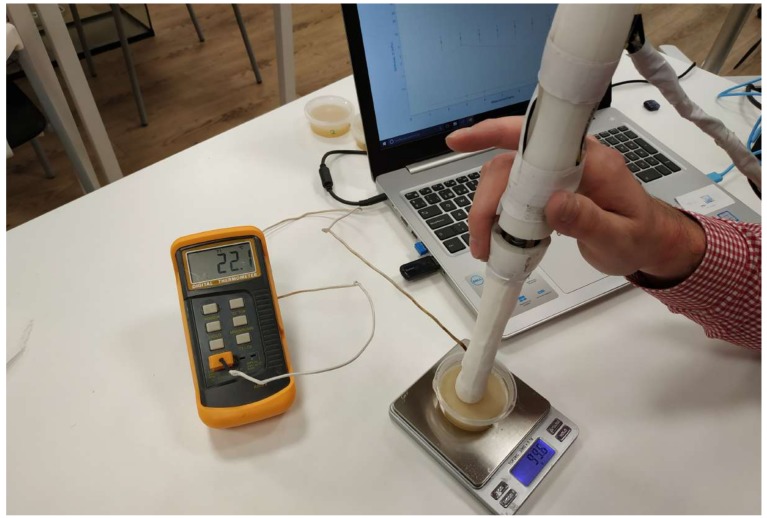
Picture of the experimental setup of TWE measurements. The phantoms were positioned on a balance to control the pressure applied.

The phantoms were tested when they reached the laboratory temperature (22 ± 1 °C and a scale was employed to quantify the applied pressure during the measurement (see Figure 5). The pressure employed (100 ± 5 g) was chosen according to a previous experience using a normal testing procedure [21]. Each of the five bilayer phantoms was measured in three different areas by an in-lab designed and prototyped probe capable of generating, receiving and analyzing torsional waves. The probe [21,49] comprises (1) an emitter, made of polylactic acid disk (PLA), whose rotational movement is due to an electromechanical actuator, (2) a receiving part composed of two PLA rings elements to reduce sensitivity to dilatational waves and an array of piezoceramic elements, and (3) a casing with geometrical and material selection to control the mechanical cross-talk.

The propagated torsional wave is a burst composed of a 1-cycle sinusoid of frequency 1000 Hz with 10x averaging to increase the strength of the signal relative to noise. For each gelatine phantom, five measurements were performed at three different points, with the objective of reconstructing the viscoelastic parameters with mean and standard deviation. The torsional sensor receives a signal in terms of voltage that is representative of the interaction with the different layers of the phantom.

### 2.4. Kelvin-Voigt FDTD Propagation Model

**Figure 6:**
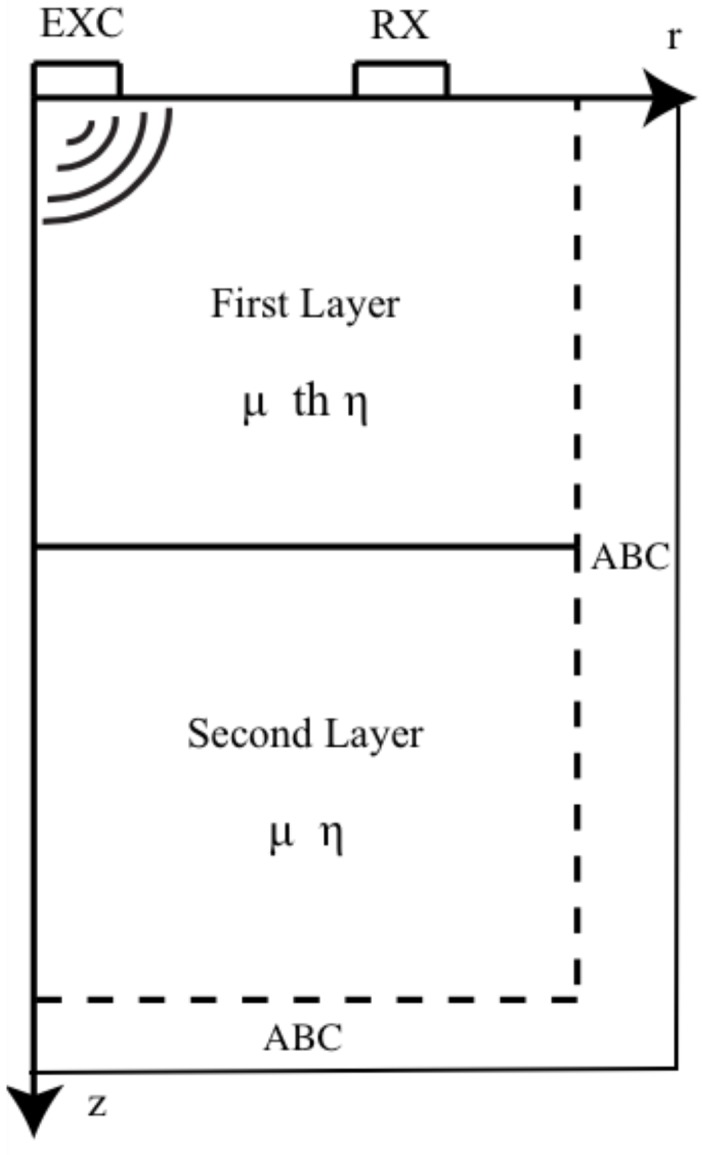
Two dimensional finite difference time domain scheme.

With the aim of inferring the viscoelastic properties of the phantoms using the PIP (explained in detail in Section 2.6), a FDTD method has been proposed in order to simulate the torsional wave propagation through the tissue-mimicking phantoms. The FDTD formulation was developed in cylindrical coordinates since torsional waves propagate axisymmetrically from the center of the probe [21]. As discussed above, with the objective of simulating the nature of the cervical tissue, two layers have been taken into account in the numerical simulations (see Figure 6).

One of the most used viscoelastic constitutive laws for modeling shear wave propagation in tissue is the KV model. Following the conclusions obtained by [21], the KV model is the one that best simulates the behavior of cervical tissue under the propagation of torsional waves.

The equations that govern the torsional wave propagation through the phantom for the KV model have been deduced taking into account the equations of motion, the kinematic relation, and the constitutive equations [28]:

(4) $$\left\{\begin{array}{c} \rho {\dot{v}}_{\theta }=\frac{\partial \sigma_{r\theta }}{\partial r}+\frac{2}{r}\sigma_{r\theta }+\frac{\partial \sigma_{\theta z}}{\partial z}\\ {\dot{\sigma }}_{r\theta }=\mu \left[\frac{\partial v_{\theta }}{\partial r}-\frac{v_{\theta }}{r}\right]+\eta \left[\frac{\partial {\dot{v}}_{\theta }}{\partial_{r}}-\frac{{\dot{v}}_{\theta }}{r}\right]\\ {\dot{\sigma }}_{\theta z}=\mu \frac{\partial v_{\theta }}{\partial z}+\eta \frac{\partial {\dot{v}}_{\theta }}{\partial z} \end{array}\right.$$

where ρ denotes the phantom density, μ and η (KV parameters) the shear elasticity and viscosity respectively, *r*, θ and *z* the cylindrical components, *v* the particle velocity and σ the stress tensor.

The system of equations was simplified by neglecting all the normal components and solely leaving the deviatoric (torsional) components. According to the work carried out by Orescanin et al. [50], this simplification did not seem to affect the results due to the low level of normal pressure generated by the exciter, in this case, the torsional probe.

The time-staggering approach used in this work consist of computing all stress, strain, and displacement at the same time interval, according to previous work carried out by Orescanin et al. [50]. Time and space were uniformly sampled, with a=iΔr/2 and b=jΔz/2 for integers a,b and space step of discretization Δr and Δz. Shear stiffness and viscosity have been introduced into the model by setting their values at the grid points of the discretized space domain. The selected values of the parameters used for the numerical FDTD simulations are summarized in Table 5.

**Figure 7:**
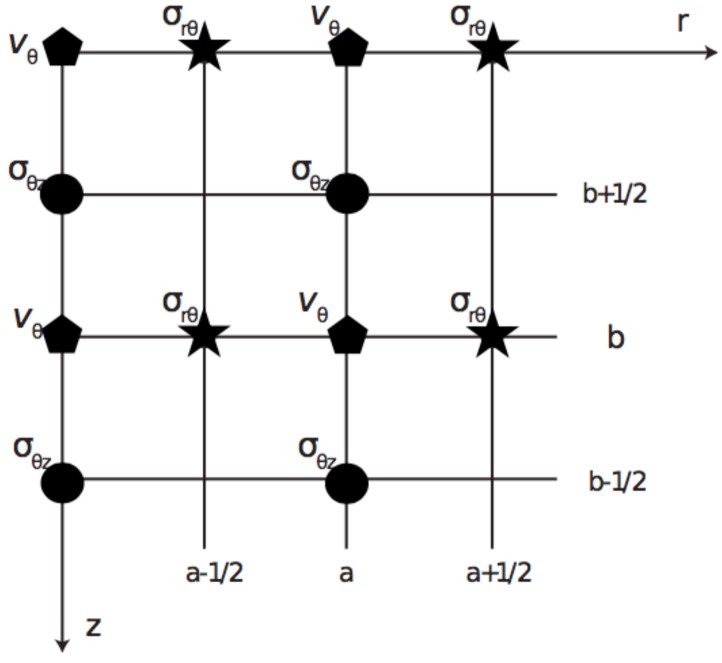
Staggered grid discretization showing the locations of variables. Velocity ($v_{\theta }$) and stresses ($\sigma_{r\theta },\sigma_{\theta z}$).

The system of Equations (4) was discretized according to Taylor series expansions [51], For derivatives with respect to one of the spatial variables (*r* or *z*), a first-order accurate centered scheme finite difference discretization has been employed (see Figure 7).

Where *f* is an arbitrary function within the domain of interest, *t* denotes the time, *x* the spatial variable *r* or *z* and Δx the spatial step for *r* or *z*.

The same centered finite difference scheme was chosen for first order time derivative (see Equations (5) and (6)):

(5) $$\frac{\partial f(x,t)}{\partial x}{|}_{x_{i},t_{n}}=\frac{f\left(x_{i+\frac{1}{2}},t_{n}\right)-f\left(x_{i-\frac{1}{2}},t_{n}\right)}{\Delta x}+O\left(\Delta x^{2}\right)$$

(6) $$\frac{\partial f(x,t)}{\partial t}{|}_{x_{i},t_{n+1}}=\frac{f\left(x_{i},t_{n+1}\right)-f\left(x_{i},t_{n}\right)}{\Delta t}+O\left(\Delta t\right)$$

**Figure 8:**
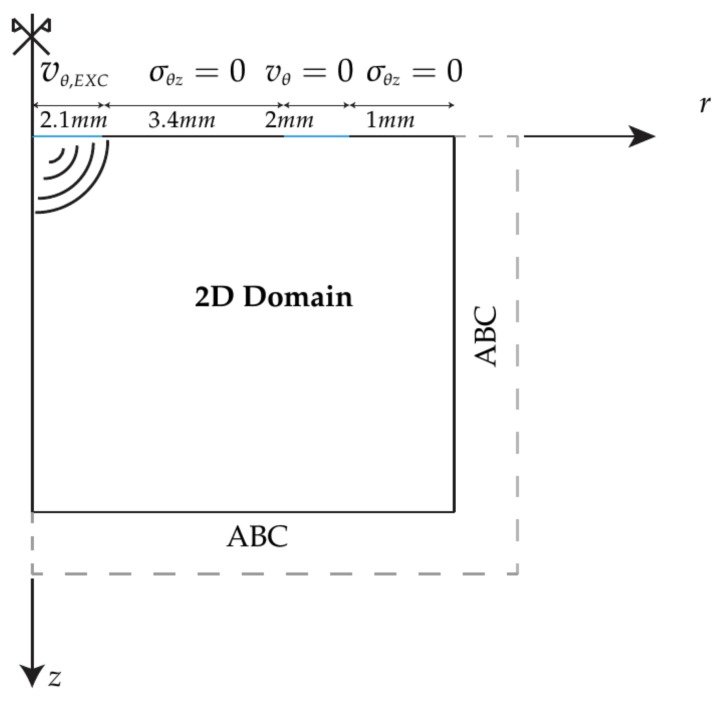
Spatial distribution of the boundary conditions of the model. 2D domain surrounded by absorbing boundary conditions, excitation, reception ($v_{\theta }=0$) and free surface conditions. Dimensions of the emitter, receiver, emitter-receiver and receiver-ABC.

The boundary conditions used in the 2D space were (Figure 8): at the surface of the tissue-mimicking phantom the excitation source, the absence of the shear stress on the surface air-phantom ($\sigma_{\theta z}\left(r,0,t_{n}\right)=0),$ the absence of velocity ($v_{\theta }\left(r_{reception},0,t_{n}\right)=0)$ in the grid point on the reception place due to the pressure applied between the receiver of the probe and the gelatine phantom and finally, the Absorbing Boundary Conditions (ABC). The ABC consist in a set of absorbing elements whose attenuation factor is leaded by an exponential law. The attenuation law ensures the reduction of reflections, thus simulating an infinite boundary condition.

The dimensions of the emitter, receiver, emitter-receiver and receiver-ABC are shown in Figure 8.

The average stress $\sigma_{\theta z}$ was obtained on the reception points. This received stress must be converted into voltage terms taking into account the layers of the receiver [21] in order to compare it with the experimental signal received by the torsional sensor (in terms of voltage). Therefore, knowing the different layers of the receiver, PLA ring-piezoelectric element (NCE51), the voltage on the piezoelectric element is approximately the stress on the tissue by a correction factor $\alpha_{c}$.

(7) $$\frac{Voltage^{NCE51}}{\sigma_{\theta z}^{phantom}}=\alpha_{c}=\text{transmission Phantom-PLA}\;\cdot \;\text{transmission PLA-NCE51}\;\cdot \;\text{factor Stress-Voltage}$$

The transmission coefficient in terms of stresses between the phantom and PLA is calculated through the shear impedance of both media (see Table 6),

(8) $$T_{Phantom-PLA}=\frac{2\;\cdot \;Z_{PLA}}{Z_{Phantom}+Z_{PLA}}=1.98$$

where $Z=c_{s}\;\cdot \;\rho$ is the shear impedance, $c_{s}$ the torsional wave speed and ρ the density of the medium.

The transmission coefficient in terms of stresses between the PLA and NCE51 and the factor stress-voltage are taken from literature [52].

(9) transmission PLA-NCE51=0.78

(10) $$\text{factor Stress-Voltage}=1.8275\cdot 10^{-4}$$

### 2.5. Viscoelastic Parameters Reconstruction from SWE Measurements

The KV viscoelastic parameters were acquired by fitting the dispersion shear wave speed curve obtained after analyzing Verasonics measurements (see Section 2.2.2). The adjustment that was performed employs an inverse problem using a combination of genetic algorithms and quasi-Newton type optimization algorithms. The complex shear moduli for the KV model is,

(11) $$G^{*KV}\left(\omega \right)=G^{\prime }\left(\omega \right)+iG^{\prime \prime }\left(\omega \right)=\mu +i\omega \eta$$

where ω is the angular frequency. The relationship between the shear wave speed ($c_{s}$) and the complex shear modulus (Equation (2)) was employed to fit the experimental data.

### 2.6. Probabilistic Inverse Problem (PIP)

We propose the technique based on a PIP with logical inference framework to evaluate the most plausible parameters of the physical model for the particular case of characterizing a viscoelastic material [26] using a KV approach. Then, the information-theoretic inverse problem framework is applied, describing the process of parametrization, the operation with discrete observation data of signals, and its extension to probabilistic parameter optimization.

The values of the search range of the parameters have been taken according to scientific evidence that are showed in [36,37], and developed in the experimental setup under the consideration of a set of phantoms previously manufactured.

To derive the effectiveness of the PIP method, the following inverse problem is solved. The outcomes are the constitutive KV viscoelastic mechanical parameters of the phantoms evaluated.

The requested constitutive equation is defined as linear viscoelasticity that additively combines strains from consistency shear modulus of first and second layer, $\mu_{first}^{E}$ and $\mu_{second}^{E},$ respectively, shear viscoelastic parameter η and thickness. The search range of these parameters are detailed in Table 7.

Note that for each sample, the same percentage of oil has been used (main responsible of the shear viscosity), this is due to the larger effect that the shear stiffness causes on the received signal with respect to the shear viscosity, in addition to the reduction of the number of parameters that are reconstructed with the PIP, which results in a reduction in the computational cost.

To solve the PIP by assuming known or unknown viscoelastic constants, the linear viscoelastic model described previously is programmed using,

(12) $$f=kf^{0}\int_{M}f\left(M\right)dM=kI$$

where M are the model parameters, $f^{0}$ is a source from experimental observations of the system and the constant *k* [53]. The integral I,

(13) $$I=\int_{M}e^{-J(M)}dM$$

(14) $$\begin{array}{c} \begin{array}{c} J=-\frac{1}{2}\int \sum_{i,j=1}^{N_{i}}\left(o_{i}\left(t,M\right)-o_{i}^{o}\left(t\right)\right){\left(C_{ij}^{o}\;+C_{ij}^{m}\right)}^{\;-1}\left(o_{j}\left(t,M\right)-o_{j}^{o}\left(t\right)\right)dt \end{array} \end{array}$$

is approximated computationally by a standard Montecarlo sampling where *J* corresponds to a misfit function between synthetic signals from the KV model $\left(o_{i}\right)$ and experimental signals (observations) ($o_{i}^{o}$), being $C_{ij}^{o}$ and $C_{ij}^{m}$ the covariance matrices that represent the error noise of the observations and model respectively, and f(M) the classical probability density which approximates the integral of any integrand f(x) that depends on the parameters *x* over a parameter subspace Ω using,

(15) $$\int_{\Omega }f\left(x\right)=\frac{1}{N}\sum_{n=1}^{N}f\left(x_{i}\right)$$

where the integrand f(x) is assessed at *N* random points $x_{i}\in \Omega$ called samples. The accurate of the algorithm is defined by the number of samples, they have been chosen as $N=2^{14}$ points. This integral is uniquely calculated at the computation of the model.

## 3. Results

### 3.1. KV Parameters Reconstruction from SWE Measurements

**Figure 9:**
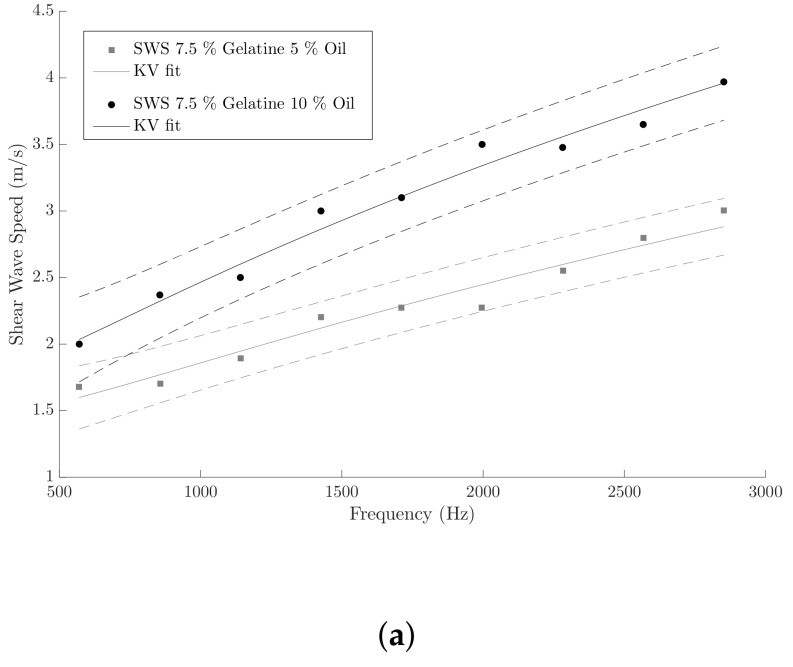

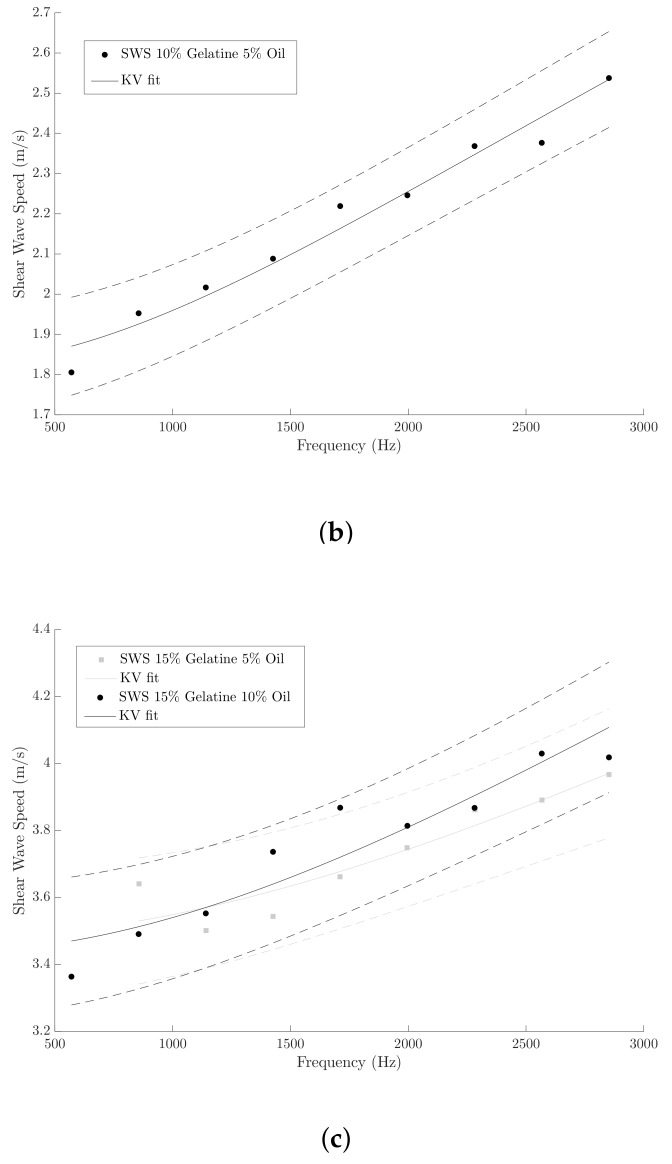
Dispersion curve for each batch of gelatine (shear wave speed data points acquired from Verasonics). The curves for Kelvin-Voigt model (solid black lines) and 95% confidence intervals (dashed lines) are shown. (**a**) Batches 7.5% gelatine 5% oil and 7.5% gelatine 10% oil; (**b**) Batch 10% gelatine 5% oil; (**c**) Batches 15% gelatine 5% oil and 15% gelatine 10% oil.

**Figure 10:**
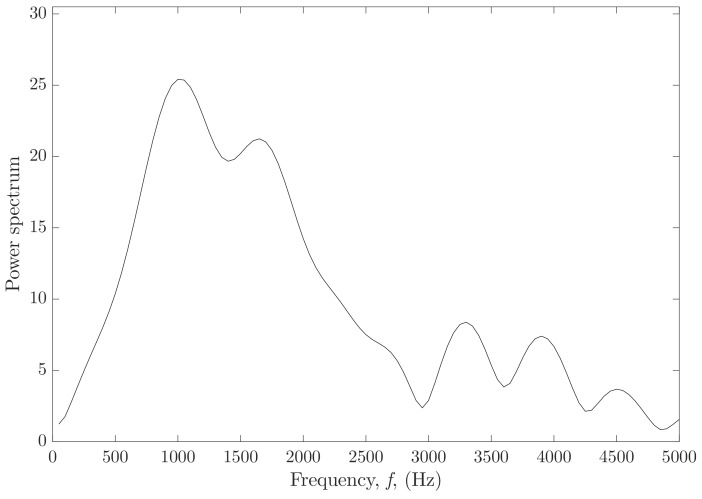
Power spectrum of the shear wave tracked for 7.5% gelatine and 10% oil batch (data acquired from Verasonics).

Shear wave speeds (dots), obtained after analyzing the data of Verasonics (see Section 2.2.2), as a function of frequency are depicted in Figure 9. The range of frequencies for each of the plots was selected according to the distribution of power into frequency components composing the shear wave signals (see Figure 10). The solid black lines are the optimal fits from the KV model for each single-layer phantom and the dashed lines are 95% confidence intervals. The KV parameters for each fitted curve are shown in Table 8. Mean and standard deviation values were estimated from three independent measurements with the SWE technique.

### 3.2. KV Parameters Reconstruction from TWE Measurements

**Figure 11:**
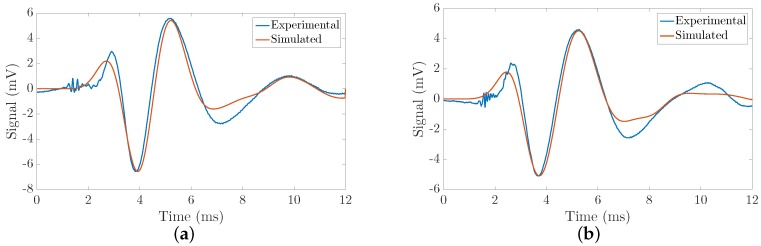
Examples of the fit of the experimental and simulated signals: Kelvin-Voigt model, time domain. (**a**) Phantom 1; (**b**) Phantom 5.

After performing the probabilistic inverse problem that mainly consists of comparing the experimental signals (acquired from TWE measurements) with those obtained from the FDTD KV model (Section 2.4), the inferred parameters are presented in Table 9. Shear elasticity, shear viscosity and the thickness of the first layer were reconstructed after solving the PIP. Mean and standard deviations values were calculated from the three independent measurements made in each specimen. Examples of the fit of experimental and simulated signals for phantoms 1 and 5 are shown in Figure 11.

### 3.3. Validation of the Kelvin-Voigt Parameters Reconstruction Method

The following table (Table 8) summarizes the values obtained with TWE and SWE for each batch of gelatine. The mean and standard deviation of the shear elasticity and shear viscosity are shown.

**Figure 12:**
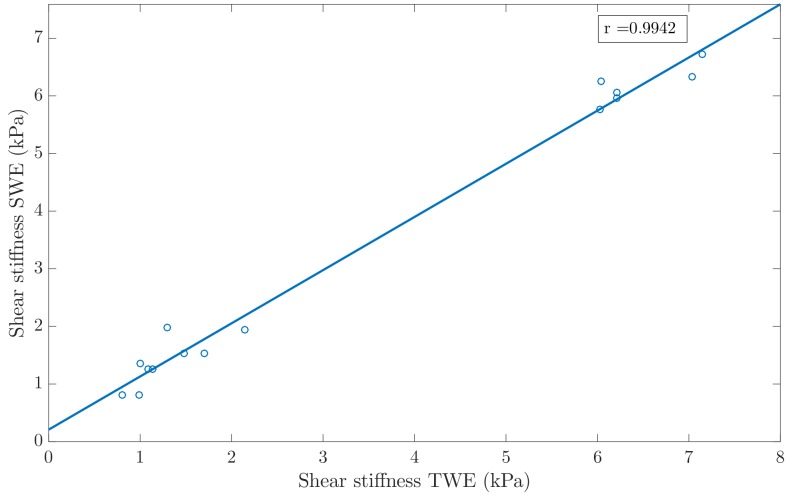
Pearson’s correlation between shear stiffness measured with TWE and shear stiffness obtained with SWE. Pearson correlation coefficient *r* = 0.9942.

**Figure 13:**
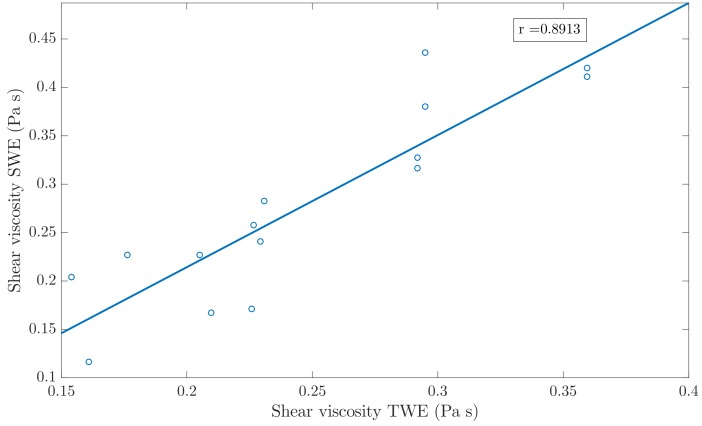
Pearson’s correlation between shear viscosity measured with TWE and shear viscosity obtained with SWE. Pearson correlation coefficient r=0.8913.

In order to study the degree of agreement between the reconstructed KV viscoelastic parameters with TWE and SWE, a Student’s T-test and a Pearson’s correlation study were carried out. Overall, the T-test yielded values higher than 0.05, which indicates that the sets of values reconstructed by both techniques are not significantly different (see Table 10). The Pearson’s correlation studied showed a high degree of agreement between the shear stiffness values obtained from both techniques (see Figure 12), with a Pearson value of r=0.9942. In the case of the shear viscosity, the degree of agreement was lower (see Figure 13), with a Pearson value of r=0.8913.

**Figure 14:**
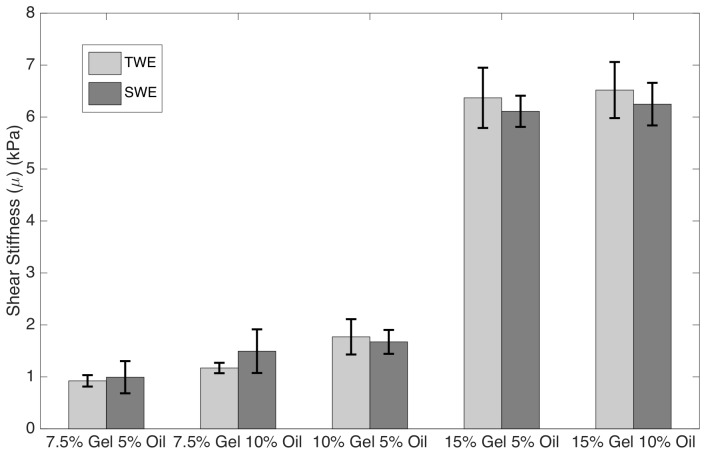
Mean and standard deviations of the shear elasticity for each batch of gelatine. Light gray bars represent TWE measurements whilst those in dark gray show SWE measurements.

**Figure 15:**
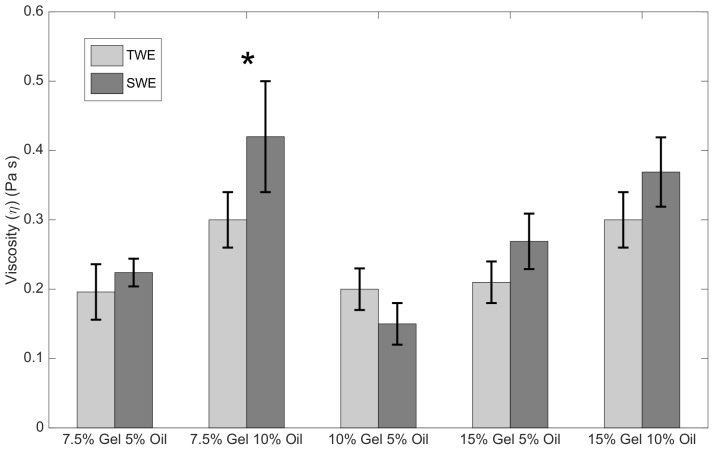
Mean and standard deviations of the shear viscosity for each batch of gelatine. Light gray bars represent TWE measurements whilst those in dark gray are SWE measurements (* *p*-value < 0.05).

Figure 14 and Figure 15 show the mean and standard values of shear elasticity and shear viscosity respectively for each batch of gelatine and elastography technique.

## 4. Discussion

The main purpose of this study is to propose and experimentally validate the results of a new method for the reconstruction of the viscoelastic parameters of cervical tissue-mimicking phantoms by the TWE technique. The TWE technique, developed by our group, aims to locally measure the mechanical parameters of the cervix, so they can be correlated with the different stages of the cervix maturing during pregnancy [21]. The previously developed reconstruction approach used is based on a TOF procedure. This approach does not take into account the fact that the waves detected on the cervix surface are altered by the mechanical response of both the epithelial and connective tissue layers of the cervix. In fact, the provided reconstructed parameter is the apparent shear wave speed measured at the surface of the cervix, which is linked to the combined mechanical response of the two tissue layers in an unknown manner. The presented reconstruction approach is based on a PIP procedure, which uses a forward model of the propagation of torsional waves in a bilayer axisymmetric cervix-like medium (see Figure 6), numerically solved by a self-developed FDTD algorithm. This way to solve the inverse problem provides the thickness of the epithelial layer, thus reconstructing separately the shear elastic modulus and the shear viscosity of each cervical layer. Furthermore, the probabilistic framework has been employed rather than the deterministic one to solve the limitations of noise in the measurements performed, heterogeneity in the properties of the sample, and even the fact that the model used to simulate its behavior is an approximation of reality.

The mechanical behavior of the cervix is viscoelastic, as most of the soft biological tissue. A viscoelastic KV constitutive law was implemented into the wave propagation model, as the KV model was found to be the simplest model in terms of a number of parameters that provides the best approximation to the mechanical behavior of ex vivo cervical tissue in our previous characterization study [21].

Five ad hoc oil-in-gelatine phantoms were fabricated to test the new reconstruction technique. The phantoms were composed of two layers to resemble the epithelial and connective cervical structure, labeled as first and second layers, respectively (see Figure 2). Different proportion of gelatine and oil were used for fabricating each layer and each phantom, so that different viscoelastic parameters where achieved (see Table 2).

The resulting viscoelastic parameters of each layer, i.e., the shear elastic modulus and the shear viscosity, fell within the range of values observed in the literature. In this study, the range obtained for the shear elasticity reconstructed parameter for the second layer (connective layer) is within the range of values estimated by Carlson et al. [11] (4.45–12.67 kPa). As far as shear viscosity in the same layer is concerned, the range obtained in this study is also in good agreement with the value found in the work performed by Peralta et al. [35] (0.26 Pa · s). To our knowledge, there are no references regarding shear elasticity and shear viscosity in the epithelial layer.

Two thickness values for the first layer were selected, 0.5 and 1 mm, which is in concordance with the values observed in the study performed by Patton et al. [36]. The thickness of the second layer was kept at 15 mm, shorter than the values found for the connective tissue layer in the literature, and sufficiently large to fully attenuate reflections from the bottom side of the phantom. Reconstructed values using the TWE technique in conjunction with the PIP approach are shown in Table 9. It can be claimed, without not much mistake, that the thickness of each phantom was satisfactorily reconstructed. Inferred thickness values were very close to the expected ones, keeping the error of reconstruction below 21%, and the standard deviation between 0.11 and 0.38 mm.

Characterization of the different oil-in-gelatine batches was needed in order to analyze the quality of the reconstruction of the viscoelastic parameters. This characterization was carried out by a well-established technique, such SWE, in this case, performed by a Verasonics system. Homogeneous phantoms were fabricated from the same oil-in-gelatine batches used before for producing the cervix-like phantoms. The propagation of the shear waves generated by the ARF yielded a shear wave dispersion speed curve for a broad range of frequency, with maximum energy between 500 and 3000 Hz (see Figure 10). This range of frequency was in agreement with the experimental SWE study in ex vivo porcine cornea [54]. As concluded by the authors, tissue targets with direct contact, such as the case of this ex vivo study, or for the ex vivo porcine cornea study, allow a higher range of frequency when using SWE ARF-based applications, compared to deep organs, such as breast, liver or prostate [54]. Oil-in-gelatine mixtures with the lower proportion of gelatine, 7.5%, and the higher proportion of oil, 10%, showed higher dispersion, i.e., steeper shear wave speed curves. Moreover, the 95% confidence intervals in this curve were also wider compared with the rest of the curves, around ±0.45 m/s against ±0.2 m/s averaged for the rest of the curves. On the contrary, mixtures with the higher proportion of gelatine, 15%, and the lower proportion of oil, 5%, showed less dispersion with a flatter variation of values in the shear wave curves. The value of the viscoelastic parameters of the different oil-in-gelatine mixtures was extracted by fitting the theoretical expression of the shear wave speed according to the KV model (a combination of Equations (2) and (11)) to the values obtained in the SWE tests (see Table 8). A direct comparison between the values obtained by both the TWE and the SWE techniques is also shown in Table 8.

The reconstruction of the viscoelastic parameters by using the TWE technique with the new proposed inversion method was validated against the SWE technique by using two approaches, a Student’s T-test and a Pearson’s correlation study. T-test results were shown in Table 10, in terms of the *p*-value for the shear elasticity and the shear viscosity from each oil-in-gelatine batch. Most of the *p*-values obtained were above 0.05, which can be considered to represent a not significant difference between the parameters reconstructed by the two techniques. Only the shear viscosity for the batch containing 7.5% of gelatine and 10% oil showed a lower *p*-value of 0.042. Nevertheless, the reconstruction of this parameter by the SWE also showed the higher range of variability, i.e., the higher standard deviation. The explanation for this low *p*-value can be associated to both the high dispersion effect of this oil-in-gelatine batch and the higher variability in the SWE measurements. Further tests should be carried out in high dispersive oil-in-gelatine phantoms in order to clarify the origin of the low correlation value.

Results from the Pearson’s correlation study were shown in Figure 12 and Figure 13, for the reconstruction of the shear stiffness and the shear viscosity parameters, respectively. As can be observed in the Figure 12 and Figure 13, the correlation between results from both techniques, the TWE and the SWE were high, specially for the shear stiffness, which yielded a Pearson’s correlation coefficient r=0.9942. The Pearson’s correlation coefficient for the shear viscosity was lower, with r=0.8913. This may be due to the larger effect that shear elastic modulus has on the torsional wave received compared to that produced by the shear viscosity. Furthermore, the reconstructions were more similar, for both the shear elasticity and shear viscosity when excluding the most dispersive batch (that made of 7.5% gelatine and 10% oil).

In general, by the obtained results from the T-test and the Pearson’s correlation study, it can be concluded that the viscoelastic parameters reconstructed by the TWE technique in conjunction with the new inversion approach are similar to those provided by the SWE technique. Therefore, it can be concluded that this new inversion method is validated for its use in the TWE technique. In order to study the performance of the new inversion method in a scenario closer to the final medical application, future work must be carried out on experimental studies using ex vivo cervical tissue samples.

## 5. Conclusions

In this work, the results of a new method based on a Probabilistic Inverse Problem (PIP) for the reconstruction of the viscoelastic parameters of cervical tissue-mimicking phantoms by the TWE technique were presented and experimentally validated. Five ad-hoc oil-in-gelatine phantoms were fabricated with different gelatine batches, simulating the anatomy of the cervical tissue composed mainly by the epithelial and connective layer, to test the new reconstruction technique. On the one hand, a probabilistic approach was employed that reconstruct the KV viscoelastic parameters by comparing the results obtained from TWE technique with the synthetic signals from the FDTD KV model. On the other hand, the characterization for each batch of gelatines was performed by SWE measurements. The validation of the method was carried out by comparing the KV parameters reconstructed from the PIP with those inferred from the shear wave speed curve obtained with SWE measurements. Finally, the degree of agreement between both techniques was tested using a Student’s T-test and a Pearson’s correlation study. As a conclusion, it can be confirmed that the viscoelastic parameters reconstructed by the TWE technique in conjunction with the PIP approach were in good agreement with those obtained by SWE technique. Future research lines must be carried out on experimental studies using ex vivo cervical tissue samples in order to test the performance of the new method.

## Figures and Tables

**Table 1:** Ingredients of the gelatine solution for the tissue-mimicking phantoms. The amount of gelatine and oil was varied for each batch (percentage specified in Table 2).

Tissue-Mimicking Phantom Constituents	
Ingredient	Supplier, Type
Gelatine	Fisher Chemical, Gelatine General purpose grade
Formalin (0.24 cc)	Sigma Aldrich, Formaldehyde sol. 37% wt in $H_{2}O$
K-Sorbate (1.62 g)	Alfa Aesar, Potassium sorbate, 99%
Lubricating Oil	50501 TDI 5W40
Surfactant (0.5 g)	Sodium dodecyl sulfate, ACS reagent, ≥99%
$H_{2}O$ (100 mL)	Laboratory distilled water

**Table 2:** Percentages of gelatine and oil wt/wt, as well as the thickness (Th) of the two layers for each of the five phantoms.

Phantoms	Layer	Gelatine (%)	Oil (%)	Th (mm)
1	First layer	7.5	5	1
	Second layer	15	5	15
2	First layer	10	5	1
	Second layer	15	5	15
3	First layer	7.5	5	1
	Second layer	10	5	15
4	First layer	7.5	10	1
	Second layer	15	10	15
5	First layer	7.5	5	0.5
	Second layer	15	5	15

**Table 3:** Properties of the L11−5v Verasonics transducer.

Number of elements	128
Pitch (mm)	0.3
Elevation focus (mm)	18
Sensitivity (dB)	−52 ± 3

**Table 4:** SWEI acquisition parameters for L11−5v Verasonics transducer.

Parameter	L11−5v
Push frequency (MHz)	4.8
Track frequency (MHz)	5.6
Push duration (cycles)	1000
Pulse repetition interval (μs)	100
Excitation Voltage (V)	28
Focal distance (mm)	20

**Table 5:** Values of the parameters for the simulations by using the FDTD model.

Parameter	Description	Value
Δr	r spatial step	75 μm
Δz	z spatial step	75 μm
Δt	time interval	1 μs
$t_{T}$	total time of simulation	12 ms
$n_{ABC}$	number of ABC elements	100

**Table 6:** Shear impedance for each medium [38].

Medium	$\rho (\mathrm{kg}/m^{3})$	$c_{s}$ (m/s)	*Z*$\mathrm{kg}/(m^{2}$ s))
**Phantom**	1000	2	2000
**PLA**	1180	200	236,000

**Table 7:** The range of the parameters implemented for the FDTD model [36,37].

Kelvin-Voigt Model	
Parameter	Search Range
$\mu_{first}$	0–20 kPa
$\mu_{second}$	0–50 kPa
η	0–5 Pa · s
th	0–2000 μm

**Table 8:** Kelvin Voigt parameters from TWE versus Verasonics for each batch of gelatine.

	TWE		SWE	
	μ± std (kPa)	η± std (Pa · s)	μ± std (kPa)	η± std (Pa · s)
7.5% Gelatine 5% Oil	0.923 ± 0.11	0.196 ± 0.04	0.993 ± 0.31	0.224 ± 0.02
7.5% Gelatine 10% Oil	1.17 ± 0.10	0.30 ± 0.04	1.494 ± 0.42	0.420 ± 0.08
10% Gelatine 5% Oil	1.77 ± 0.34	0.20 ± 0.03	1.673 ± 0.23	0.150 ± 0.03
15% Gelatine 5% Oil	6.37 ± 0.58	0.21 ± 0.03	6.111 ± 0.30	0.269 ± 0.04
15% Gelatine 10% Oil	6.52 ± 0.54	0.30 ± 0.04	6.249 ± 0.41	0.369 ± 0.05

**Table 9:** Kelvin Voigt parameters reconstruction from TWE. Data from the first and second layer is shown (shear elasticity and shear viscosity), including the thickness of the first layer for each of the five phantoms.

Phantoms	Layer	μ (kPa)	η (Pa · s)	Th (mm)
1	First layer	1.14 ± 0.15	0.20 ± 0.03	1.00 ± 0.12
	Second layer	6.31 ± 0.62		-
2	First layer	1.43 ± 0.17	0.22 ± 0.02	0.79 ± 0.11
	Second layer	6.02 ± 0.55		-
3	First layer	0.72 ± 0.045	0.19 ± 0.05	1.15 ± 0.38
	Second layer	2.12 ± 0.52		-
4	First layer	1.17 ± 0.10	0.30 ± 0.04	0.87 ± 0.19
	Second layer	6.52 ± 0.54		-
5	First layer	0.91 ± 0.16	0.20 ± 0.04	0.56 ± 0.23
	Second layer	6.79 ± 0.57		-

**Table 10:** Shear elasticity and shear viscosity measurements comparison between TWE and SWE for each batch of gelatine. *p*-Value obtained from the Student’s T-test was the metric used for this comparison. (* *p*-value < 0.05).

	*p*-Value	
Batch	μ	η
7.5% Gelatine 5% Oil	0.365	0.1640
7.5% Gelatine 10% Oil	0.134	0.041 *
10% Gelatine 5% Oil	0.262	0.0551
15% Gelatine 5% Oil	0.274	0.0553
15% Gelatine 10% Oil	0.263	0.067

## Abbreviations

The following abbreviations are used in this manuscript:

TWE	Torsional Wave Elastography
PIP	Probabilistic Inverse Problem
KV	Kelvin-Voigt
FDTD	Finite Difference Time Domain
ABC	Absorbing Boundary Conditions
SWE	Shear Wave Elastography
TOF	Time-Of-Flight
ARF	Acoustic Radiation Force
MTL	Multiple Tracking Location
IQ	In-Phase and Quadrature
USTB	Ultrasound Toolbox
FFT	Fast Fourier Transform
PLA	Polylactic Acid
